# Supplementary material for: Self-assembly coupled to liquid-liquid phase separation
Source: PLoS Comput Biol. 2023 May 15;19(5):e1010652. doi: 10.1371/journal.pcbi.1010652 (PMC10212142; doi:10.1371/journal.pcbi.1010652)
Supplement: S1 Text — Section A: The kinetics of subunits and assemblies partitioning between the compartment and background. Section B: Scaling estimates of elongation timescales. Section C: Scaling estimates of maximum assembly speedup. Section D: Analysis for nnuc = 2. (PDF) [file pcbi.1010652.s001.pdf]

Supporting Information for: Self-Assembly Coupled to Liquid-Liquid Phase Separation

Michael F. Hagan[1,*] and Farzaneh Mohajerani[1]

[1] *Martin A. Fisher School of Physics, Brandeis University, Waltham, Massachusetts, United States of America*

(Dated: May 1, 2023)

S1 Text Section A: The kinetics of subunits and assemblies partitioning between the compartment and background

In this section we consider the kinetics of subunits and assemblies partitioning between the compartment and background. We will see that under most cases the rate of subunits partitioning into the compartment will be fast relative to assembly timescales, and thus in the main text we will develop scaling estimates based on a quasi-equilibrium assumption for the ratio of subunit concentrations in the compartment and background.

For a single spherical compartment, the rate for intermediates of size n to enter the compartment is given by $k_{\text{DL}}(n)\rho_1^{\text{bg}}$, with the diffusion-limited adsorption rate constant $k_{\text{DL}}(n) = 4\pi R_{\text{c}} D_n$, with R_{c} the radius of the compartment, and D_n the diffusion constant for the intermediate. The dependence of diffusion constant on particle size is complex due to crowding and active processes (e.g. [1, 2]). The diffusion coefficient for individual proteins is typically $\sim 0.1 D_0$, with $D_0 = 100 \mu\text{m}^2/s$ the value in dilute solution. For simplicity, we assume a form

$$D_n = r_{\text{diff}} D_0 n^{-\gamma} \tag{S1}$$

with r_{diff} the ratio of the diffusion coefficient for a protein monomer in the cell relative to dilute solution, and the factor n^{γ} gives the scaling of the hydrodynamic radius with size for the assembly, which depends on its geometry. In the results below we will typically set $r_{\text{diff}} = 0.1$ and $\gamma = 1/2$, where the latter is the scaling for a capsid-like assembly. In general the results will not depend (even quantitatively) on these values. However, if the diffusion of large intermediates and assembly products is sufficiently suppressed within a cell, the distribution of assembly sizes will be under kinetic control even at long times.

* hagan@brandeis.edu

Finally, to allow for the possibility of arrested or microphase separation, we could consider n_c compartments with radius $R_\mathrm{c} = \left(\frac{3}{4\pi}\frac{V_\mathrm{c}}{n_\mathrm{c}}\right)^{1/3}$.

Putting these considerations together results in a diffusion limited rate constant

$$k_\mathrm{DL}(n) = 4\pi n_\mathrm{c} R_\mathrm{c} r_\mathrm{diff} n^{-\gamma} D_0. \tag{S2}$$

For simplicity we assume diffusion rates are uniform throughout the background and compartment.

At equilibrium, the flux of subunit adsorption into the compartment must be balanced by the outward flux, leading to the detailed balance condition on the desorption rate constant (k_desorb) relative to adsorption, $k_\mathrm{DL}/k_\mathrm{desorb} = K_\mathrm{c}$. Thus, if we assume a fixed subunit concentration far from the compartment ρ_1^bg, the kinetics of adsorption into the compartment follows

$$\begin{aligned}\frac{d\rho_n^\mathrm{c}}{dt} &\equiv \mathcal{D}_n^\mathrm{dom} = \frac{1}{V_\mathrm{c}} k_\mathrm{DL}(n)\left(\rho_n^\mathrm{bg} - \rho_n^\mathrm{c}/K_\mathrm{c}^n\right)\\ \frac{d\rho_n^\mathrm{bg}}{dt} &\equiv \mathcal{D}_n^\mathrm{bg} = -\frac{V_\mathrm{c}}{V_\mathrm{bg}}\frac{d\rho_n^\mathrm{c}}{dt}.\end{aligned} \tag{S3}$$

To get a feeling for the numbers, let us again consider a single compartment with radius $R_\mathrm{c} = 1$ μm in a cell with radius $R_\mathrm{cell} = 10$ μm (so $V_\mathrm{r} = 10^{-3}$), and typical diffusion coefficient for protein in the cytoplasm $D = 10$ $\mu\mathrm{m}^2$/s ($r_\mathrm{diff} = 0.1$), which gives a diffusion-limited rate for monomers of $k_\mathrm{DL} = 4\pi \times 10^{10}$ $\mathrm{nm}^3/s = 7.6 \times 10^{10}/\mathrm{M}\cdot\mathrm{s}$. In the limit $x_\mathrm{selec} \gg 1$ so that essentially all assembly occurs within the compartment, the characteristic timescale for a significant fraction of subunits to reach the compartment is given from Eq. (S3) as

$$\tau_\mathrm{diff} \sim V_\mathrm{c}/k_\mathrm{DL} \approx 30\mathrm{s}. \tag{S4}$$

Under most conditions that we consider below this will be significantly faster than the characteristic assembly nucleation timescale and hence diffusion timescales can be neglected. However, under conditions of extremely strong partitioning into the compartment and thus high local concentrations and rapid nucleation, the ability of a single compartment to accelerate assembly will be limited by this diffusion timescale. But, allowing for multiple compartments would reduce this timescale.

S1 Text Section B: Scaling estimates of elongation timescales

For the model we are focusing on, a linear nucleation and growth process with f independent of intermediate size, we can specifically estimate the elongation timescale by the mean first passage time for a biased random walk with a reflecting boundary conditions at n_nuc and absorbing boundary conditions at N, with forward and reverse hopping rates given by the subunit association and

dissociation rates respectively [3]. For early in the reaction, when $\rho_1 \approx \rho_{\mathrm{T}}$, this results in

$$\begin{aligned}\tau_{\mathrm{elong}} =& \frac{n_{\mathrm{elong}}}{f\rho_{\mathrm{T}} - b_{\mathrm{elong}}} \\ &- \frac{b_{\mathrm{elong}}}{\left(f\rho_{\mathrm{T}} - b_{\mathrm{elong}}\right)^2}\left(1 - \left(\frac{b_{\mathrm{elong}}}{f\rho_{\mathrm{T}}}\right)^{n_{\mathrm{elong}}}\right)\end{aligned} \tag{S5}$$

with $n_{\mathrm{elong}} = N - n_{\mathrm{nuc}} \approx N$. In the limit of $f\rho_{\mathrm{T}} \gg b_{\mathrm{elong}}$ Eq. S5 can be approximated to give $\tau_{\mathrm{elong}} \approx n_{\mathrm{elong}}/f\rho_{\mathrm{T}}$, or $\alpha = 1$ in Eq. 19 (main text), while for similar forward and reverse reaction rates, $f\rho_{\mathrm{T}} \approx b_{\mathrm{elong}}$, it approaches the solution for an unbiased random walk $\tau_{\mathrm{elong}} \approx n_{\mathrm{elong}}^2/2f\rho_{\mathrm{T}}$, or $\alpha = 2$. For the CNT model, since association rates are $\propto n^{1/2}$, we obtain $\alpha = 1/2$ for strongly forward-biased assembly [3].

S1 Text Section C: Scaling estimates of maximum assembly speedup

In this section we evaluate the maximum possible speedup due to LLPS accounting for the monomer depletion kinetic trap. Recalling that the minimum assembly timescale τ_{min} occurs at ρ_* when the elongation and median assembly timescales are equal, we obtain

$$\begin{aligned}\tau_{\mathrm{min}}(V_{\mathrm{r}}, K_{\mathrm{c}}) &\approx 2\tau_{\mathrm{elong}}(\rho_*(V_{\mathrm{r}}, K_{\mathrm{c}})) \\ &\cong 2\frac{A_{1/2}x_N}{Nf}\exp\left(G_{\hat{n}}/k_{\mathrm{B}}T\right)\frac{\left[\rho_*(V_{\mathrm{r}}, K_{\mathrm{c}})\right]^{-\hat{n}}}{s_{\mathrm{nuc}}(V_{\mathrm{r}}, K_{\mathrm{c}})} \\ &\cong 2N/f\rho_*(V_{\mathrm{r}}, K_{\mathrm{c}})K_{\mathrm{eff}}K_{\mathrm{c}}.\end{aligned} \tag{S6}$$

Defining the assembly speedup as $s_{\mathrm{LLPS}}(K_{\mathrm{c}}, V_{\mathrm{r}}) \equiv \tau^0/\tau(K_{\mathrm{c}}, V_{\mathrm{r}})$, our goal will be to estimate the maximum speedup possible at a given subunit concentration and compartment size by optimizing τ_{min} over K_{c}. To simplify the calculation, we assume that LLPS dominates near the maximal speedup and thus assembly primarily occurs in the compartment, so we can simplify the nucleation acceleration as $s_{\mathrm{nuc}} \approx V_{\mathrm{r}}\left(K_{\mathrm{eff}}K_{\mathrm{c}}\right)^{\hat{n}}$, the elongation time as $\tau_{\mathrm{elong}} \approx 2N/f\rho_* K_{\mathrm{eff}}K_{\mathrm{c}}$, and the median assembly time as $\tau_{1/2} \approx \frac{A_{1/2}x_N}{Nf}\exp\left(G_{\hat{n}}/k_{\mathrm{B}}T\right)\frac{\rho_*^{-\hat{n}}}{V_{\mathrm{r}}(K_{\mathrm{eff}}K_{\mathrm{c}})^{\hat{n}}}$, and $\rho_* \approx \rho_*^0/\left[V_{\mathrm{r}}\left(K_{\mathrm{eff}}K_{\mathrm{c}}\right)^{\hat{n}}\right]^{1/(\hat{n}-1)}$.

Now, we optimize over K_{c} to obtain

$$\left(K_{\mathrm{eff}}K_{\mathrm{c}}\right)^* \approx \left(\frac{\rho_*^0}{\rho_{\mathrm{T}}}\right)^{(\hat{n}-1)/\hat{n}}\frac{1}{V_{\mathrm{r}}^{1/\hat{n}}}. \tag{S7}$$

Solving for the optimal value of the partition coefficient K_{c}^* then yields

$$K_{\mathrm{c}}^* \approx \left[\frac{1 + V_{\mathrm{r}}}{\left(K_{\mathrm{eff}}K_{\mathrm{c}}\right)^*} - V_{\mathrm{r}})\right]^{-1} \tag{S8}$$

with $K_{\mathrm{c}}^* \to \infty$ for $\left(K_{\mathrm{eff}}K_{\mathrm{c}}\right)^* >= 1 + 1/V_{\mathrm{r}}$.

We then use Eq. S7 obtain the *maximum* speedup as

$$s^*_{\mathrm{LLPS}} \equiv \max_{K_\mathrm{c}} s_{\mathrm{LLPS}} \approx V_\mathrm{r}^{-1/\hat{n}} \left(\frac{\rho^0_*}{\rho_\mathrm{T}}\right)^{(\hat{n}^2-1)/\hat{n}} \tag{S9}$$

S1 Text Section D: Analysis for $n_{\mathbf{nuc}} = 2$

Assembly timescales without LLPS. To this point we have assumed the existence of a nucleation barrier and thus a critical nucleus size $n_{\mathrm{nuc}} \geq 3$ in our expression for the dependence of the nucleation rate on subunit concentration (Eq. 20 main text). For the special case of no nucleation barrier ($n_{\mathrm{nuc}} = 2$), the reaction is unregulated and reduces to the well-studied case of unregulated 1-D filament assembly. If the association rate is independent of assembly size n, assembly rapidly results in an exponential distribution of filament sizes whose mean gradually increases through coarsening (but is cut off by the maximal size N). Regulation can be restored if the association rate of the first two subunits is slow compared to subsequent association events (e.g. if the subunits must undergo a conformational change in order to assemble [4]). In this case, the median assembly time is given by $\tau^0_{1/2}(n_{\mathrm{nuc}} = 2) \approx 1/f_2 N \rho_\mathrm{T}$ with f_2 the dimerization rate constant. Equating $\tau^0_{1/2}(n_{\mathrm{nuc}} = 2) = \tau^0_{\mathrm{elong}}$ then shows that the form of the kinetics is independent of ρ_T, but the reaction behaves as if regulated when $f_2 \ll f$ with the threshold dimerization rate given by $f^0_{\mathrm{KT}} = f/N^2$. Here we have assumed that the association rate constant f is independent of size for $n > 2$ for simplicity.

Assembly timescales with LLPS. Accounting for the presence of a compartment results in

$$\tau_{1/2} \approx 1/f_2 N \rho_\mathrm{T} s_{\mathrm{nuc}} \tag{S10}$$

and τ_{elong} given by Eq. 19 in the main text. As in the case without LLPS, the threshold for monomer-starvation is independent of total subunit concentration, but the threshold dimerization rate constant is now given by

$$f_{\mathrm{KT}} = \frac{f}{N^2} \frac{s_{\mathrm{elong}}}{s_{\mathrm{nuc}}}. \tag{S11}$$

Importantly, Eq. (S11) shows that LLPS allows for the reaction to be regulated even for the case of the rate constant independent of assembly size ($f_2 = f$).

---

[1] L. Stadler and M. Weiss, Non-equilibrium forces drive the anomalous diffusion of telomeres in the nucleus of mammalian cells, New Journal of Physics **19**, 113048 (2017).

[2] B. Parry, I. Surovtsev, M. Cabeen, C. O'Hern, E. Dufresne, and C. Jacobs-Wagner, The bacterial cytoplasm has glass-like properties and is fluidized by metabolic activity, Cell **156**, 183 (2014).

[3] M. F. Hagan and O. Elrad, Understanding the Concentration Dependence of Viral Capsid Assembly Kinetics - the Origin of the Lag Time and Identifying the Critical Nucleus Size, Biophys. J. **98**, 1065 (2010).

[4] G. Lazaro and M. Hagan, Allosteric control in icosahedral capsid assembly, J. Phys. Chem. B **120**, 6306 (2016).
