# Supplementary material for: Self-assembly coupled to liquid-liquid phase separation
Source: PLoS Comput Biol. 2023 May 15;19(5):e1010652. doi: 10.1371/journal.pcbi.1010652 (PMC10212142; doi:10.1371/journal.pcbi.1010652)
Supplement: Attachment — Submitted filename: 2022-Dec-PlosComputBiol.pdf [file pcbi.1010652.s002.pdf]

PCOMPBIOL-D-22-01510

Summary:

Hagan and Mohajerani use a master equation approach to delineate the effects of liquid-liquid phase separation (LLPS) on the thermodynamics and kinetics of reversible self-assembly. The overarching theme is to decipher how self-assembly proceeds within a phase-separated compartment (or so called 'domain' by the authors) versus from a bulk solution (i.e., absence of phase separation).

There are several interesting findings in the manuscript, including: (1) the presence of the phase-separated compartment allows assembly under conditions where there is no bulk assembly; (2) LLPS increases the range of concentrations under which productive assembly occurs; (3) the bulk solution acts as a buffer that steadily provides 'subunits' (i.e., the components that self-assemble within the domain; e.g., proteins) to the domain thereby avoiding kinetic traps during the self-assembly process; (4) the degree of speedup during self-assembly increases as the volume ratio (roughly volume of the domain vs volume of the 'background'; where the background is the remaining bulk) decreases. This rate also enhances with decreasing total subunit concentration and increasing critical nucleus size.

In general, the authors are rigorous in their derivations and application of relevant theories (such as nucleation-growth models and classical nucleation theory). However, the way in which the results are framed/presented could be improved significantly. There are also several assumptions/decisions made throughout that require further justification.

Major remarks:

(1) From the title and abstract, the work seemingly probes a widespread phenomenon. However, the manuscript focuses heavily on the case of virus capsid assembly. Are there other examples where self-assembly within 'domains' is relevant? If not, perhaps the manuscript should be reframed as a study of capsid assembly in/out of domains. Otherwise, the authors should give more experimental examples where this process is relevant.

(2) The quality of the figures could be improved. Also, it would be helpful to readers if there are well-labeled graphics throughout that clearly explain the various scenarios being discussed in the text. These could be useful in defining the main parameters of the model (e.g., domains, subunits), especially in cases where *new* terminology is being introduced to discuss established ideas.

(3) It is not immediately clear what the domains and background are composed of. The authors describe a binary solvent but are there experimental analogues to this set up? How different are the subunits from the other components of the domains? Do they have to be significantly distinct for the model to be valid?

(4) What feature of the subunits is responsible for their preferential partition into the domains? This point needs to be discussed further. For example, do the subunits interact with the domain components? If that is true can the composition of the domain and the subunit concentration be regarded as independent of each other?

(5) In much of the manuscript, the authors assume that the volume ratio (approx. volume of domain vs volume of background) is very small (<<1). But also, that the subunits are a small fraction of the domain mass. Given these limits, how does the domain size compare to the size of the assembly? What are the consequences of this, in the context of the distinguishability of the domain and the assembly? Is there a minimum domain size that is required to allow productive assembly? How does this scale with the size of the subunits?

(6) On a similar front, the case where the subunits make up a large fraction of the domain mass would be perhaps more likely in biology. How can the model account for this?

(7) In Figure 2, besides broadening the concentrations for assembly, the maximum on short timescales also shifts to a slightly higher f_N. What is the significance of this?

(8) In Equation 4, wouldn't there be free subunits in the background as well? As written, only free subunits are within the domain.

(9) The concept of selectivity in the manuscript is not properly defined.

(10) The assumption that the diffusion coefficient is independent of subunit concentration seems counterintuitive since the subunits are being preferentially partitioned into the domains. How can this be reconciled?

Minor remarks:

(1) The sentence "*The subunits are immersed in a binary solvent (which could be proteins, polymers, nucleic acids, etc.) which is in a state of phase coexistence"* is confusing. As written, it appears that the binary solvent is made up of proteins and instead of the subunits could be proteins.

(2) There is no figure 5B.
