# Supplementary material for: Self-assembly coupled to liquid-liquid phase separation
Source: PLoS Comput Biol. 2023 May 15;19(5):e1010652. doi: 10.1371/journal.pcbi.1010652 (PMC10212142; doi:10.1371/journal.pcbi.1010652)
Supplement: Attachment — Submitted filename: Response to reviewers 2023.03.26.pdf [file pcbi.1010652.s003.pdf]

We thank the reviewers for their careful reading of the revised manuscript and their constructive comments. We have addressed all their comments as described below.

The most significant critiques were that the biological motivation for self-assembly in LLPS droplets was not sufficiently explained and there was insufficient justification or explanation for key approximations in the model. In response to these critiques, we have significantly expanded the introduction, description of the model, and conclusions. For example, we have included examples of LLPS-coupled assembly in biology for systems other than viruses. Further, we provide greater description of the type of LLPS droplet that we are considering, its constituent components, and how the assembling subunits differ from droplet constituents. We also provide a specific motivating example of such a phase-separated droplet from rotavirus assembly. In addition, we have expanded on the model description: we have added a new schematic figure (new Fig. 2) to explain the nucleation-and-growth classical nucleation theory pathways, we explicitly list all the key assumptions and approximations, we discuss in greater detail the consequent limitations, in particular those of neglecting the possibility of malformed capsids. We also have followed the other suggestions from the reviewers to improve the quality of the manuscript. Finally, we added a “Methods” section to comply with the PLOS formatting requirement; we moved the description of the models into that section.

We present our response and associated modifications to each comment point-by-point below. We also have provided a marked-up version of the manuscript in which the corresponding changes are tracked. (We have included a marked-up version from before moving the model description to the “Methods” section, as identifying the substantive changes became too difficult after that).

Referee 1:

Summary:

Hagan and Mohajerani use a master equation approach to delineate the effects of liquid-liquid phase separation (LLPS) on the thermodynamics and kinetics of reversible self-assembly. The overarching theme is to decipher how self-assembly proceeds within a phase-separated compartment (or so called ‘domain’ by the authors) versus from a bulk solution (i.e., absence of phase separation).

There are several interesting findings in the manuscript, including: (1) the presence of the phase-separated compartment allows assembly under conditions where there is no bulk assembly; (2) LLPS increases the range of concentrations under which productive assembly occurs; (3) the bulk solution acts as a buffer that steadily provides ‘subunits’ (i.e., the components that self-assemble within the domain; e.g., proteins) to the domain thereby avoiding kinetic traps during the self-assembly process; (4) the degree of speedup during self-assembly increases as the volume ratio (roughly volume of the domain vs volume of the ‘background’; where the background is the remaining bulk) decreases. This rate also enhances with decreasing total subunit concentration and increasing critical nucleus size.

In general, the authors are rigorous in their derivations and application of relevant theories (such as nucleation- growth models and classical nucleation theory). However, the way in which the results are framed/presented could be improved significantly. There are also several assumptions/decisions made throughout that require further justification.

Major remarks:

(1) *From the title and abstract, the work seemingly probes a widespread phenomenon. However, the manuscript focuses heavily on the case of virus capsid assembly. Are there other examples where self- assembly within 'domains' is relevant? If not, perhaps the manuscript should be reframed as a study of capsid assembly in/out of domains. Otherwise, the authors should give more experimental examples where this process is relevant.*

Response: While to our knowledge the best studied examples of self-assembly in phase-separated compartments involve viruses, the phenomenon occurs in other systems as well. We have added references to some examples.

Modifications: We have added references to other examples of assembly in phase-separated compartments in the introduction (line 50):
"In addition to viruses, other examples of biological self-assembly coupled to LLPS include the formation of clatherin cages to mediate endocytosis [112]; post-synaptic densities [113] and pre-synaptic vesicles release sites (active zones) [114,115] at neuronal synases; observations that condensates can both accelerate and suppress aggregation of α-synuclein [116], and actin assembly in polypeptide coacervates [117]."

(2) *The quality of the figures could be improved. Also, it would be helpful to readers if there are well-labeled graphics throughout that clearly explain the various scenarios being discussed in the text. These could be useful in defining the main parameters of the model (e.g., domains, subunits), especially in cases where new terminology is being introduced to discuss established ideas.*

Modifications: We have added a new figure (new Fig. 2) which shows schematics that illustrate the two assembly models that we are using. We have also added descriptive labels to Figures 3, 4, and 7, and made other changes to figures in response to further reviewer comments addressed below.

(3) *It is not immediately clear what the domains and background are composed of. The authors describe a binary solvent but are there experimental analogues to this set up? How different are the subunits from the other components of the domains? Do they have to be significantly distinct for the model to be valid?*

Response: For this first study of self-assembly within phase-separated compartments, we have chosen the simplest possible model, in which the subunits are suspended in a binary solvent which phase separates into a compartment rich in one solvent component, with the background rich in the other solvent component. This is a simplification of, for example, recent in vitro experiments in which SARS-CoV-2 nucleocapsid proteins assemble in phase-separated droplets formed by the SARS-CoV-2 RNA molecules. Similarly, it is a highly simplified scenario motivated by rotavirus viroplasms in cells, in which the phase-separated compartment is formed primarily by two nonstructural proteins, NS2 and NS5. The capsid proteins, VP2 and VP6, and the viral RNA molecules preferentially partition into the compartment, but at much lower concentrations than NS2 and NS5.

Do they have to be significantly distinct for the model to be valid?

The key difference is that the primary compartment constituents remain disordered and in the liquid phase, while the assembly components form ordered (para)crystalline (but self-limited in size) structures such as a virus capsid.

Modification: We have elaborated on the motivation for our model. We also emphasize the approximations underlying the simplifications that we have made.

Line 109: "We consider a system of subunits that form self-limited assemblies with optimal size N. The subunits are immersed in a multicomponent solvent which is in a state of phase coexistence, with stoichiometry such that there are one or more small compartments rich in one (or more) solvent species coexisting with a much larger background rich in the other species. While we consider protein subunits undergoing assembly, the solvent could be comprised of proteins, nucleic acids, or other macromolecules. The distinction is that `subunits' form ordered (para)crystalline structures such as a capsid, while the primary compartment constituents remain amorphous and (for the regime we consider) in the liquid phase. Our model can be viewed as a minimal starting point motivated by biological assembly coupled to LLPS. For example, in the model proposed for rotavirus assembly, two nonstructural rotavirus proteins (NSP2 and NSP5) undergo LLPS to form a viroplasm. The capsid proteins (VP2 and VP6) partition favorably into the viroplasm due to weak multivalent interactions with NSP2 and NSP5 [94, 98, 104, 107]. Here, NSP2 and NSP5 would correspond to the primary compartment constituents, and the capsid proteins (VP2 and VP6) correspond to the assembly subunits. We note that the rotavirus genomic RNA is also driven to partition into the viroplasm through interactions between RNA binding proteins and NSP2/5, but we do not explicitly consider assembly around RNA in this work to simplify the model. Our assumption of low concentration of assembly subunits in the droplet noted above is consistent with concentrations of VP2/6 in the viroplasm that are small compared to those of NSP2/5 [94, 98, 104, 107]."

(4) *What feature of the subunits is responsible for their preferential partition into the domains? This point needs to be discussed further.*

Response: This point is discussed at the beginning of the Model section, where we introduce the partition coefficient. Please see the text starting after Line 130, above Eq. (1). In brief, the subunits are driven to partition into the compartment through weak multivalent attractive interactions with the primary constituent particles of the compartment. For example, in the case of the in vitro experiments on SARS-CoV-2 nucleocapsid protein noted in the introduction, interactions between the nucleocapsid protein and RNA drive subunits into the compartment. Similarly, in the case of rotavirus viroplasms, the capsid proteins are driven to partition into the viroplasm by interactions with the primary viroplasm components, NS2 and NS5. As noted above, we have now significantly elaborate on this in the revised version with reference to the rotavirus example.

For example, do the subunits interact with the domain components? If that is true can the

composition of the domain and the subunit concentration be regarded as independent of each other?

To simplify the calculations, we assume that the subunits remain in the dilute limit, i.e. comprising a low mole fraction of the domain constituents. This is a good approximation for example, in the case of the rotavirus viroplasms, where the domain is primarily composed of NS2 and NS5. In this limit, the domain composition is approximately independent of subunit concentration. This assumption is stated at the end of the first paragraph of the Model section (Lines 105-108).

Modifications:
(1) We have expanded the discussion of the driving force for subunits to partition into the compartment phase, making an explicit analogy with the rotavirus example (starting at Line 118).

(2) We clarify the fact that we restrict the calculations to the dilute limit of subunits, and we more strongly emphasize that based on this limit we are making the simplifying assumption that the domain composition is independent of subunit concentration. We have added the following text:

Line 127: "Our assumption of low concentration of assembly subunits in the compartment noted above is consistent with concentrations of VP2/6 in the viroplasm that are small compared to those of NSP2/5 [94, 98, 104, 107].

Line 174: "Third, we assume that the domain composition is independent of subunit concentration and assembly…. These simplifications are based on the assumption of low concentrations of subunits in the compartment and that the other compartment constituents are macromolecules that typically have equal or larger molecular weights as the subunits."

(5) *In much of the manuscript, the authors assume that the volume ratio (4pprox.. volume of domain vs volume of background) is very small (<<1). But also, that the subunits are a small fraction of the domain mass. Given these limits, how does the domain size compare to the size of the assembly? What are the consequences of this, in the context of the distinguishability of the domain and the assembly? Is there a minimum domain size that is required to allow productive assembly? How does this scale with the size of the subunits?*

Response/Modification: We have added a new subsection, '**Typical and minimal compartment sizes**' starting at Line 151, to address this question. As shown there, for a typical eukaryotic cell with radius 10 μM, and our default compartment volume ratio of 0.001, the compartment radius is $R_{\mathrm{c}} = 1$ μM. A typical protein with mass 30 kDa has a volume of about 50 nm3. Thus, even if we require the mole fraction of proteins ≤ 0.01, this allows for 8x10^5 proteins within the domain. While this number is well below the thermodynamic limit, it is large compared to a typical assembly size of 100-1000 subunits, and sufficiently large that finite number fluctuations can be neglected at least to a first approximation. Along these lines, a "minimum" domain size might be defined as the smallest domain in which an assembly of, let's say 1000 subunits, would correspond to a small fraction of 0.01, which gives $R_{\mathrm{min}} \approx 0.1$ μM. This would scale with the subunit size (or more interestingly with the size of the assembly) as $R_{\mathrm{min}} \propto \text{size}^{1/3}$, and thus is insensitive to either subunit size or assembly size.

(6) *On a similar front, the case where the subunits make up a large fraction of the domain mass would be perhaps more likely in biology. How can the model account fo this?*

As discussed above, to our knowledge it is not the typical case in biology that assembly subunits make up a large fraction of the domain mass. However, we do plan on accounting for this scenario in a later work, by explicitly modeling the dynamics of the droplet formation using standard reaction diffusion formalisms, including accounting for interactions between subunits and other droplet constituents. However, this is beyond the scope of the present, first study of assembly coupled to LLPS.

(7) *In Figure 2, besides broadening the concentrations for assembly, the maximum on short timescales also shifts to a slightly higher fN. What is the significance of this?*

Response: This is one of the important features of LLPS – in addition to making assembly more robust to parameter variations, it allows more efficient assembly. The system achieves higher concentrations in a given time scale because both nucleation and growth timescales are reduced by locally concentrating subunits within the droplet, while the overall monomer depletion rate is slowed by the fact that assembly only occurs within the droplet.

Modification: We have added the following sentences to further explain this point (Line 288): "Notice that in addition to making assembly more robust, LLPS also increases the maximum yield achievable at finite times. This increase arises because both nucleation and elongation rates can locally increase within the droplet due to the high local concentration while avoiding free subunit depletion."

(8) *In Equation 4, wouldn't there be free subunits in the background as well? As written, only free subunits are within the domain.*

Response: You are correct, there was a typo in the equation. The term in the second set of ()'s on the RHS should be the concentration of free subunits in the background.

Modification: we have fixed this typo

(9) *The concept of selectivity in the manuscript is not properly defined.*

Response: We define selectivity in the following sentence above Eq. (18) (located below Line 245 in the revised version):
"As a measure of the extent to which LLPS can spatially control assembly, we define *selectivity* as …."

Modifications:
(1)To make this definition more visible, we have added a bold paragraph heading (Line 245): "**Selectivity and spatial control over assembly.**"

(2) We now also include the symbol for selectivity in the y-axis label of Fig. 6 (Fig. 5 in the original version).

(10) *The assumption that the diffusion coefficient is independent of subunit concentration seems counterintuitive since the subunits are being preferentially partitioned into the domains. How can this be reconciled?*

Response: We expect the diffusion coefficient to be roughly independent of subunit concentration in the limit that subunits are relatively dilute. This is especially reasonable considering that the other constituents of the domain are typically equal or larger molecular weight than the subunits, and the total density of macromolecules within a droplet is not likely to depend on subunit concentration.

Modification: We have added the following sentences to explain the justification for this assumption, Line 174:
"Third, we assume that the domain composition is independent of subunit concentration and assembly. Similarly, we assume that the diffusion coefficient is independent of subunit concentration. These simplifications are based on the assumption of low concentrations of subunits in the compartment and that the other compartment constituents are macromolecules that typically have equal or larger molecular weights as the subunits."

Minor remarks:

(1) The sentence "*The subunits are immersed in a binary solvent (which could be proteins, polymers, nucleic acids, etc.) which is in a state of phase coexistence"* is confusing. As written, it appears that the binary solvent is made up of proteins and instead of the subunits could be proteins.

Modification: We have rewritten this sentence as (Line 110):
"The subunits are immersed in a multicomponent solvent which is in a state of phase coexistence, with stoichiometry such that there are one or more small compartments rich in one (or more) solvent species coexisting with a much larger background rich in the other species. While we consider protein subunits undergoing assembly, the solvent could be comprised of proteins, nucleic acids, or other macromolecules. The distinction is that `subunits' form ordered (para)crystalline structures such as a capsid, while the primary compartment constituents remain amorphous and (for the regime we consider) in the liquid phase. "

(2) *There is no figure 5B.*

Response: Thank you for identifying this error.

Modification: We edited the sentence referring to Fig. 5B to refer to Fig. 6.

Referee 2:
Reviewer #2: This is an interesting theoretical development that addresses the thermodynamic and dynamic ramifications for the modulation of viral-capsid-like assembly in cellular compartments that

maintain a higher concentration of subunits. The results are suitable for publication in PLoS CB, but revision is necessary to clarify/address the following issues:

1. The authors elect to use "domain" to describe the compartment that maintain a higher concentration of subunits. This term is not ideal if the authors aim to frame their investigation in the context of recent advances in the study of biomolecular condensates (which is clearly the case judging from the large number of references in this area that the authors included). These condensates are usually referred to as "compartments". In this very field that the authors aim to contextualize their work, "domain" usually refers to protein domains. So the authors' terminology will not help facilitate understanding by readers who are familiar with the biomolecular condensate terminology. I suggest that the authors to change "domain" to "compartment" (they did use "compartmentalization" on p.12 of the manuscript). Failing that, they should at least state clearly at the outset that their "domain" is equivalent to intracellular membraneless biomolecular compartments.

Response: This is a good suggestion. We have changed from "domain" to "compartment" throughout the manuscript.

Modification: We have changed all uses of the word "domain" to "compartment". Correspondingly, all superscripts or subscripts that were previously written as "dom" are now written as "c".

2. Though it is not stated explicitly in the text, the formulation in Eqs.13-14 indicates that subunits can only be added one-by-one in the authors' model. In other words, associations of, say, two n=2 complexes is postulated to be impossible (because there are no such terms in Eqs.13-14). Is this the case? If so, this limitation has to be justified. Does it have an experimental basis in viral capsid assembly? Irrespective of whether viral capsid assembly obeys this rule, the authors should discuss (and/or show additional calculation) what will happen when this limitation is lifted.

Response: We have made the simplifying assumption that subunits only add one-by-one. Thank you for pointing out that we failed to note this. This assumption is justified in two ways: (1) Previous simulations with this model that did allow for binding of subunit dimers demonstrate no qualitative difference in comparison to binding of only individual subunits (see the supplemental material of Hagan and Elrad, Biophys. J. 2010). (2) Dynamical particle-based computer simulations which make no assumptions about assembly pathways show that most assembly events involve association of individual subunits at the dilute conditions typical of productive assembly reactions. There are examples in which assembly of higher-order oligomers are important, but these correspond either to high concentrations or assembly reactions in which there are activation barriers during the elongation phase (after formation of the critical nucleus) to addition of individual subunits. By construction, such barriers in the elongation phase are not present in this simple model.

Although it is straightforward to extend the model to allow for binding of dimers and other oligomers as we did in Hagan and Elrad 2010, given that it will not qualitatively change results, we feel that it is not worth making the paper longer to include additional results on this.

Modification: We now explicitly state the simplifying assumption that subunits only add one-by-one, and explain the justification for this assumption (Line 166):
"We have made several assumptions to simplify the models, based on previous work (e.g [28, 29, 39, 41, 138–140]). First, we assume that there is only one 'average' intermediate structure for each size n.

Second, we assume that only individual subunits can associate to or dissociate from an intermediate. This assumption is based on the fact that particle-based computer simulations show that, at the dilute conditions typical of productive assembly reactions, most assembly events involve association of individual subunits [138], and that extending Eq. (13) to allow for binding of higher-order oligomers does not qualitatively change the results (see the supplemental material of Ref. [138]).”

3. Notation problem: fN is used to denote fraction of completed assembly in Fig.2 and Fig.3 but is also used (on the same page) to represent associate rate constant for the n=N state in Eq.14. This situation should be rectified. Authors should use different symbols for different quantities in the same paper, especially when different meanings of the same symbols are invoked so close to each other in the text.

Response: Thank you for catching this oversight. In fact f_N need not be in Eq. 14 (now Eq. 2 in the revised version) since it is zero by construction (sorry for this typo). To avoid possible confusion however, we have changed the symbol for fraction of completed assembly to the typical symbol for a mole fraction, x_N.

Modifications:
(1) We removed f_N from Eq. 14 (now Eq. 2)
(2) We changed the mole fraction capsid symbol from f_N to x_N.

4. Additional information should be provided in Fig.3 to show corresponding data for ρT > 1µM (by adding a figure or adding parts to this figure or simply by adding curves to the existing figure). This is important for illustrating the decreases seen for the red curves in Fig.2 for larger ρT , which is an interesting finding. To make this correspondence clearer, vertical dashed lines should be added to indicate t = 1 day time point (24 hr) along the horizontal scales of these plots.

Response: We already show results for ρT=4 µM in Fig. 3A of the original manuscript (now Fig. 4A in the revised version), please see the purple curve (second from the bottom at the final time point). We also highlight the fact that this result illustrates the nonmonotonic dependence of finite-time assembly on total concentration at the end of the first paragraph in the section ‘Master equation results’ (subsubsection ‘Assembly kinetics and yields’, Line 255). Based on the fact that the reviewer did not see this, we now try to better emphasize this important point.

Modifications:
(1) We have edited the three (previously 2) paragraphs starting with (Line 255) “**Assembly kinetics and yields.** Figs. 3 and 4 show the effect of LLPS on assembly kinetics…” to better emphasize this point and the underlying physics.

(Line 263): “Importantly, the rate of capsid production is nonmonotonic with respect to ρ_{T} --- yields of complete capsids are suppressed for the highest concentration shown ($\rho_{\mathrm{T}} = 4\ \mu$M) by the *monomer starvation* kinetic trap arising from depletion of free subunits before capsids finish assembling. These results are discussed further in section ‘Assembly timescales without LLPS’ and Refs. [41, 138]. This kinetic trapping effect is responsible for the low values of x_N at high concentrations for finite-time results in Fig. 3.

(Line 270): “Figs. 4B and 3A show how the assembly kinetics is changed by LLPS…. Notice that in addition to making assembly more robust, LLPS also increases the maximum yield achievable at finite times. This

increase arises because both nucleation and elongation rates can locally increase within the droplet due to the high local concentration while avoiding free subunit depletion."

(2) We have added the vertical dashed lines at the 1-day time point as suggested.

5. It is important to emphasize that the kinetic traps mentioned in the present work are caused by depletion of subunits but not "malformed assembly" (mentioned in page 12). Malformed assemblies can also cause kinetic traps (as their disassembly requires an energetic cost). This matter should be commented upon with a clarification about the basic difference between these two types of kinetic traps.

Response: We agree that malformed assembly kinetic traps are also important, and we have significantly expanded discussion of them. We now note that we are neglecting this possibility during presentation of the model, and we discuss the implications of this simplification in more detail in the Conclusions.

We are currently studying this possibility using particle-based simulations. Our results indicate that while malformed traps occur at extremely high local concentrations within the droplet and thus do set a threshold to the possible achievable speed up, for reasonable orientational specificity of the subunits this threshold concentration is large enough that it does not qualitatively change the results compared to what is described in the present manuscript. However, it would be too much to include these results in the present manuscript.

Modifications:
(1) (Line 183): We have added the following sentences in the discussion of model simplifications: "The most important simplification is that we neglect the possibility of malformed (off-pathway) structures. While this is a good assumption under productive assembly conditions particularly when subunit-subunit interactions have high orientational specificity, malformed structures can lead to kinetic traps at high concentrations or binding affinities [32–34, 36–42, 141–143]. This effect will be considered by performing particle-based simulations in a future work, and we discuss its implications in the Conclusions section."

(2) (Line 509): We have expanded discussion of malformed assemblies in the Conclusions as follows: "Importantly, the rate equation models and scaling estimates considered here do not account for kinetic traps resulting from malformed assemblies, which can arise when subunits bound with incorrect geometries do not have time to anneal before becoming locked into place by association of additional subunits (e.g. [11, 32, 39, 41, 42, 141, 142, 149, 150]). Since association rates increase with concentration, we anticipate that malformed assemblies will occur above a threshold local concentration within the compartment, thus limiting the maximum speed up provided by LLPS. This threshold concentration increases with the geometric specificity of the subunit-subunit interactions. Thus, for sufficiently specific interactions the results described here will not qualitatively change when accounting for malformed structures --- there will be a significant range of local concentrations, and thus assembly speed up, before either the malformed structure for monomer starvation kinetic trap set in. We will explore the effects of malformed assemblies on LLPS-coupled assembly in a future work."

6. More specific examples should be provided in the concluding discussion about what real biophysical process this formulation is expected to be applicable to. What kind of viral capsid? Which intracellular compartments (domains) are the likely locations for viral capsid assembly?

Response: In the original manuscript we presented phase-separated compartments that viruses generate and assemble within as example systems that our models could apply to. We now discuss a viral system that particularly motivates our work, rotavirus assembly, in some detail in the presentation of the model. Based on the reviewer comment, we have also expanded on our discussion of these systems in the conclusions as well.

Modifications:
(1) (Line 50): We present examples of systems other than viruses in which assembly occurs within phase-separated compartments and thus could be used to test our predictions:
"In addition to viruses, other examples of biological self-assembly coupled to LLPS include the formation of clatherin cages to mediate endocytosis [112]; post-synaptic densities [113] and pre-synaptic vesicles release sites (active zones) [114,115] at neuronal synases; observations that condensates can both accelerate and suppress aggregation of α-synuclein [116], and actin assembly in polypeptide coacervates [117]."

(2) In the presentation of the model, we describe rotavirus viroplasms in detail (Line 118):
"Our model can be viewed as a minimal starting point motivated by biological assembly coupled to LLPS. For example, in the model proposed for rotavirus assembly, two nonstructural rotavirus proteins (NSP2 and NSP5) undergo LLPS to form a viroplasm. The capsid proteins (VP2 and VP6) partition favorably into the viroplasm due to weak multivalent interactions with NSP2 and NSP5 [94, 98, 104, 107]. Here, NSP2 and NSP5 would correspond to the primary compartment constituents, and the capsid proteins (VP2 and VP6) correspond to the assembly subunits. We note that the rotavirus genomic RNA is also driven to partition into the viroplasm through interactions between RNA binding proteins and NSP2/5, but we do not explicitly consider assembly around RNA in this work to simplify the model. Our assumption of low concentration of assembly subunits in the compartment noted above is consistent with concentrations of VP2/6 in the viroplasm that are small compared to those of NSP2/5 [94, 98, 104, 107]."

(3) In the conclusions, we have added further discussion of biological examples, in the paragraph that begins with (Line 493):
"Such phase-separated compartments appear to be ubiquitous in cells, and as noted in the introduction, assembly of diverse structures such as clatherin cages, actin filaments, and neuronal synapses can occur within compartments. The systems which most directly inspired this work are the phase-separated compartments generated during viral infections (e.g. virus factories, replication sites, Negri bodies, inclusion bodies, or viroplasms…"

7. The authors clearly aim to be rather comprehensive in their citing literature in the Introduction. In this regard, references for several key advances in the theoretical study of biomolecular condensates are missing, which should be included in the revised version of this manuscript, as follows: Lin et al., Phys Rev Lett 117:178101 (2016) (first theory for sequence-dependent biomolecular phase separation) should be included around ref.[50], and Lin & Chan, Biophys J 112:2043-2046 (2017); Amin et al., J Phys Chem B 124:6709-6720 (2020) – two theoretical works that address the relationship between single-chain and two-chain association properties and multiple-chain phase separation propensity – should be included around ref.[63].

Response: Thank you for bringing these key articles to our attention
Modification: We have added the references as suggested.

8. Likely typographical errors:
(i) 3rd line below Eq.10: “Kdom → 0” should be “Kdom → 1”?

(ii) Eq.A3: subscript n is missing in the two diffusion coefficient. Judging from these typos, there are likely others – this referee did not do an exhaustive check. The authors are strongly encouraged to double-check to make sure that their notation is clear and consistent and without omissions and typos.

Response/Modifications: Thank you, we fixed these typos

We have checked the revised manuscript exhaustively for typos, but we apologize for any others that we have missed and very much appreciate the referees pointing out any that they do see.
