# Supplementary material for: Self-assembly coupled to liquid-liquid phase separation
Source: PLoS Comput Biol. 2023 May 15;19(5):e1010652. doi: 10.1371/journal.pcbi.1010652 (PMC10212142; doi:10.1371/journal.pcbi.1010652)
Supplement: Attachment — Submitted filename: Response letter.pdf [file pcbi.1010652.s004.pdf]

We have edited figure 1 so that the text is more visible. In particular, we’ve changed the font color to black for all text, and we’ve surrounded it in a light gray text box to a visibility.

We also made several small edits to the mean text to address two typos, and to eliminate one unneeded approximation from an equation.
